# Supplementary material for: Mathematical modeling reveals ferritin as the strongest cellular driver of dietary iron transfer block in enterocytes
Source: PLoS Comput Biol. 2025 Mar 7;21(3):e1012374. doi: 10.1371/journal.pcbi.1012374 (PMC11918390; doi:10.1371/journal.pcbi.1012374)
Supplement: S1 Text — (PDF) [file pcbi.1012374.s001.pdf]

Supplement file S1 Text

Masison & Mendes (2025) “Mathematical modeling reveals ferritin as the strongest cellular driver of dietary iron transfer block in enterocytes”

Differential equation model

$$\frac{\mathrm{d}\left([\text{FT-cage}]\cdot V_{\text{cell}}\right)}{\mathrm{d}t} = -V_{\text{cell}}\cdot\left(\text{FT-kdeg}\cdot[\text{FT-cage}]\right) + V_{\text{cell}}\cdot\left(\text{FT-a}\cdot\left(1-\frac{[\text{IRPs}_{\text{active}}]^{n_{(\text{FT expression})}}}{K_{(\text{FT expression})}^{n_{(\text{FT expression})}}+[\text{IRPs}_{\text{active}}]^{n_{(\text{FT expression})}}}\right)\right)$$

$$\frac{\mathrm{d}\left([\text{core}]\cdot V_{\text{cell}}\right)}{\mathrm{d}t} = +2\cdot V_{\text{cell}}\cdot\left(\frac{\mathrm{k1}_{(\text{FT core formation})}\cdot[\text{DFP}]\cdot[\text{core}]}{\mathrm{k2}_{(\text{FT core formation})}+[\text{DFP}]}\cdot\frac{\mathrm{k3}_{(\text{FT core formation})}^{n_{(\text{FT core formation})}}}{\mathrm{k3}_{(\text{FT core formation})}^{n_{(\text{FT core formation})}}+[\text{core}]^{n_{(\text{FT core formation})}}}\cdot\frac{4300^{m_{(\text{FT core formation})}}-\text{atoms-per-cage\_transient}^{m_{(\text{FT core formation})}}}{4300^{m_{(\text{FT core formation})}}}\right)$$
$$+4\cdot V_{\text{cell}}\cdot\left(\mathrm{k1}_{(\text{FT nucleation})}\cdot[\text{DFP}]^2\cdot[\text{FT-cage}]\cdot\frac{L+\text{rN}}{24+\text{rN}}\cdot\frac{\text{ki}_{(\text{FT nucleation})}^{n_{(\text{FT nucleation})}}}{\text{ki}_{(\text{FT nucleation})}^{n_{(\text{FT nucleation})}}+[\text{core}]^{n_{(\text{FT nucleation})}}}\right)$$
$$-V_{\text{cell}}\cdot\left(\text{FT-kdeg}\cdot\frac{[\text{core}]}{[\text{FT-cage}]}\cdot[\text{FT-cage}]\right)$$

$$\frac{\mathrm{d}\left([\text{DFP}]\cdot V_{\text{cell}}\right)}{\mathrm{d}t} = -V_{\text{cell}}\cdot\left(\frac{\mathrm{k1}_{(\text{FT core formation})}\cdot[\text{DFP}]\cdot[\text{core}]}{\mathrm{k2}_{(\text{FT core formation})}+[\text{DFP}]}\cdot\frac{\mathrm{k3}_{(\text{FT core formation})}^{n_{(\text{FT core formation})}}}{\mathrm{k3}_{(\text{FT core formation})}^{n_{(\text{FT core formation})}}+[\text{core}]^{n_{(\text{FT core formation})}}}\cdot\frac{4300^{m_{(\text{FT core formation})}}-\text{atoms-per-cage\_transient}^{m_{(\text{FT core formation})}}}{4300^{m_{(\text{FT core formation})}}}\right)$$
$$-V_{\text{cell}}\cdot\left(\mathrm{k1}_{(\text{FT Fe reduction})}\cdot[\text{DFP}]\right)$$
$$+V_{\text{cell}}\cdot\left(\frac{\text{kcat}_{(\text{FT Fe oxidation})}\cdot\frac{H+\text{rO}_{(\text{FT Fe oxidation})}}{24+\text{rO}_{(\text{FT Fe oxidation})}}\cdot[\text{FT-cage}]\cdot[\text{LIP}]^{n_{(\text{FT Fe oxidation})}}}{\text{Km}_{(\text{FT Fe oxidation})}^{n_{(\text{FT Fe oxidation})}}+[\text{LIP}]^{n_{(\text{FT Fe oxidation})}}}\right)$$
$$-2\cdot V_{\text{cell}}\cdot\left(\mathrm{k1}_{(\text{FT nucleation})}\cdot[\text{DFP}]^2\cdot[\text{FT-cage}]\cdot\frac{L+\text{rN}}{24+\text{rN}}\cdot\frac{\text{ki}_{(\text{FT nucleation})}^{n_{(\text{FT nucleation})}}}{\text{ki}_{(\text{FT nucleation})}^{n_{(\text{FT nucleation})}}+[\text{core}]^{n_{(\text{FT nucleation})}}}\right)$$

$$\frac{\mathrm{d}\left([\text{LIP}]\cdot V_{\text{cell}}\right)}{\mathrm{d}t} = +2\cdot V_{\text{cell}}\cdot\left(\mathrm{k1}_{(\text{FT Fe reduction})}\cdot[\text{DFP}]\right)$$
$$-2\cdot V_{\text{cell}}\cdot\left(\frac{\text{kcat}_{(\text{FT Fe oxidation})}\cdot\frac{H+\text{rO}_{(\text{FT Fe oxidation})}}{24+\text{rO}_{(\text{FT Fe oxidation})}}\cdot[\text{FT-cage}]\cdot[\text{LIP}]^{n_{(\text{FT Fe oxidation})}}}{\text{Km}_{(\text{FT Fe oxidation})}^{n_{(\text{FT Fe oxidation})}}+[\text{LIP}]^{n_{(\text{FT Fe oxidation})}}}\right)$$
$$+V_{\text{apical}_{\text{mem}}}\cdot\left(\text{kcat}_{(\text{DMT1 iron transport})}\cdot[\text{DMT1}]\cdot\frac{\left(\frac{[\text{Fe}_{\text{lumen}}]}{K_{(\text{DMT1 iron transport})}}\right)^{n_{(\text{DMT1 iron transport})}}}{1+\left(\frac{[\text{Fe}_{\text{lumen}}]}{K_{(\text{DMT1 iron transport})}}\right)^{n_{(\text{DMT1 iron transport})}}}\right)$$
$$+\left(\mathrm{k1}_{(\text{Fe basal uptake})}\cdot[\text{Fe}_{\text{blood}}]\right)$$
$$+V_{\text{cell}}\cdot\left(\text{FT-kdeg}\cdot\frac{[\text{core}]}{[\text{FT-cage}]}\cdot[\text{FT-cage}]\right)$$
$$-V_{\text{BLM}}\cdot\left(\text{kcat}_{(\text{FPN iron transport})}\cdot[\text{FPN}_{\text{active}}]\cdot\frac{\left(\frac{[\text{LIP}]}{K_{(\text{FPN iron transport})}}\right)^{n_{(\text{FPN iron transport})}}}{1+\left(\frac{[\text{LIP}]}{K_{(\text{FPN iron transport})}}\right)^{n_{(\text{FPN iron transport})}}}\right)$$

$$\frac{\mathrm{d}\left([\text{DMT1}]\cdot V_{\text{apical}_{\text{mem}}}\right)}{\mathrm{d}t} = -V_{\text{apical}_{\text{mem}}}\cdot\left(k_{(\text{DMT1 endocytosis free})}\cdot[\text{DMT1}]\right)$$
$$+V_{\text{apical}_{\text{mem}}}\cdot\left(\mathrm{k1}_{(\text{DMT1\_i fusion})}\cdot[\text{DMT1}_{\text{vesicular}}]\right)$$
$$-\left(\text{kcat}_{(\text{DMT1 endocytosis modified})}\cdot[\text{DMT1}]\cdot\frac{\left(\frac{[\text{LIP}]}{K_{(\text{DMT1 endocytosis modified})}}\right)^{n_{(\text{DMT1 endocytosis modified})}}}{1+\left(\frac{[\text{LIP}]}{K_{(\text{DMT1 endocytosis modified})}}\right)^{n_{(\text{DMT1 endocytosis modified})}}}\right)$$

$$\frac{\mathrm{d}\left([\text{DMT1}_{\text{vesicular}}]\cdot V_{\text{apical}_{\text{mem}}}\right)}{\mathrm{d}t} = +V_{\text{apical}_{\text{mem}}}\cdot\left(k_{(\text{DMT1 endocytosis free})}\cdot[\text{DMT1}]\right)$$
$$-V_{\text{apical}_{\text{mem}}}\cdot\left(\mathrm{k1}_{(\text{DMT1\_i fusion})}\cdot[\text{DMT1}_{\text{vesicular}}]\right)$$
$$+\left(\text{kcat}_{(\text{DMT1 endocytosis modified})}\cdot[\text{DMT1}]\cdot\frac{\left(\frac{[\text{LIP}]}{K_{(\text{DMT1 endocytosis modified})}}\right)^{n_{(\text{DMT1 endocytosis modified})}}}{1+\left(\frac{[\text{LIP}]}{K_{(\text{DMT1 endocytosis modified})}}\right)^{n_{(\text{DMT1 endocytosis modified})}}}\right)$$

$$\frac{\mathrm{d}\left([\text{Fe}_{\text{blood}}]\cdot V_{\text{lower}}\right)}{\mathrm{d}t} = -\left(\mathrm{k1}_{(\text{Fe basal uptake})}\cdot[\text{Fe}_{\text{blood}}]\right)$$
$$+\left(\left(\mathrm{k1}_{(\text{paracellular fe transport})}\cdot[\text{Fe}_{\text{lumen}}]-\mathrm{k2}_{(\text{paracellular fe transport})}\cdot[\text{Fe}_{\text{blood}}]\right)\right)$$
$$-V_{\text{lower}}\cdot\left(\mathrm{k1}_{(\text{body sequestration})}\cdot[\text{Fe}_{\text{blood}}]\right)$$
$$+V_{\text{BLM}}\cdot\left(\text{kcat}_{(\text{FPN iron transport})}\cdot[\text{FPN}_{\text{active}}]\cdot\frac{\left(\frac{[\text{LIP}]}{K_{(\text{FPN iron transport})}}\right)^{n_{(\text{FPN iron transport})}}}{1+\left(\frac{[\text{LIP}]}{K_{(\text{FPN iron transport})}}\right)^{n_{(\text{FPN iron transport})}}}\right)$$

$$\frac{\mathrm{d}\left([\text{IRPs}_{\text{active}}]\cdot V_{\text{cell}}\right)}{\mathrm{d}t} = +V_{\text{cell}}\cdot\left(\mathrm{k1}_{(\text{IRPs activation})}\cdot[\text{IRPs}_{\text{inactive}}]\right)$$
$$-V_{\text{cell}}\cdot\left(\text{ka}_{(\text{IRPs degradation})}\cdot[\text{IRPs}_{\text{active}}]\cdot[\text{LIP}]\right)$$

$$\frac{\mathrm{d}\left([\text{FPN}_{\text{internalized}}]\cdot V_{\text{BLM}}\right)}{\mathrm{d}t} = +\left(\mathrm{k1}_{(\text{FPN-inactivation})}\cdot[\text{FPN}_{\text{active}}]\cdot\frac{[\text{Fe}_{\text{blood}}]^{n_{(\text{FPN-inactivation})}}}{\mathrm{k3}_{(\text{FPN-inactivation})}^{n_{(\text{FPN-inactivation})}}+[\text{Fe}_{\text{blood}}]^{n_{(\text{FPN-inactivation})}}}\right)$$
$$-\left(\mathrm{k1}_{(\text{FPN-activation})}\cdot[\text{FPN}_{\text{internalized}}]\right)$$

$$\frac{\mathrm{d}\left([\text{IRPs}_{\text{inactive}}]\cdot V_{\text{cell}}\right)}{\mathrm{d}t} = -V_{\text{cell}}\cdot\left(\mathrm{k1}_{(\text{IRPs activation})}\cdot[\text{IRPs}_{\text{inactive}}]\right)$$
$$+V_{\text{cell}}\cdot\left(\text{ka}_{(\text{IRPs degradation})}\cdot[\text{IRPs}_{\text{active}}]\cdot[\text{LIP}]\right)$$

$$\frac{\mathrm{d}\left([\text{Fe}_{\text{lumen}}]\cdot V_{\text{upper}}\right)}{\mathrm{d}t} = -V_{\text{apical}_{\text{mem}}}\cdot\left(\text{kcat}_{(\text{DMT1 iron transport})}\cdot[\text{DMT1}]\cdot\frac{\left(\frac{[\text{Fe}_{\text{lumen}}]}{K_{(\text{DMT1 iron transport})}}\right)^{n_{(\text{DMT1 iron transport})}}}{1+\left(\frac{[\text{Fe}_{\text{lumen}}]}{K_{(\text{DMT1 iron transport})}}\right)^{n_{(\text{DMT1 iron transport})}}}\right)$$
$$-\left(\left(\mathrm{k1}_{(\text{paracellular fe transport})}\cdot[\text{Fe}_{\text{lumen}}]-\mathrm{k2}_{(\text{paracellular fe transport})}\cdot[\text{Fe}_{\text{blood}}]\right)\right)$$

$$\frac{\mathrm{d}\left([\text{body}_{\text{fe}}]\cdot V_{\text{lower}}\right)}{\mathrm{d}t} = +V_{\text{lower}}\cdot\left(\mathrm{k1}_{(\text{body sequestration})}\cdot[\text{Fe}_{\text{blood}}]\right)$$

$$\frac{\mathrm{d}\left([\text{FPN}_{\text{active}}]\cdot V_{\text{BLM}}\right)}{\mathrm{d}t} = -\left(\mathrm{k1}_{(\text{FPN-inactivation})}\cdot[\text{FPN}_{\text{active}}]\cdot\frac{[\text{Fe}_{\text{blood}}]^{n_{(\text{FPN-inactivation})}}}{\mathrm{k3}_{(\text{FPN-inactivation})}^{n_{(\text{FPN-inactivation})}}+[\text{Fe}_{\text{blood}}]^{n_{(\text{FPN-inactivation})}}}\right)$$
$$+\left(\mathrm{k1}_{(\text{FPN-activation})}\cdot[\text{FPN}_{\text{internalized}}]\right)$$

$$\text{atoms-per-cage\_transient} = \frac{\text{core}.\mathit{ParticleNumber}}{\text{FT-cage.ParticleNumber}}$$

$$L = 24-H$$
