# Supplementary material for: Mathematical modeling reveals ferritin as the strongest cellular driver of dietary iron transfer block in enterocytes
Source: PLoS Comput Biol. 2025 Mar 7;21(3):e1012374. doi: 10.1371/journal.pcbi.1012374 (PMC11918390; doi:10.1371/journal.pcbi.1012374)
Supplement: S2 Text — (PDF) [file pcbi.1012374.s002.pdf]

Supplement file S2 Text

Masison & Mendes (2025) “Mathematical modeling reveals ferritin as the strongest cellular driver of dietary iron transfer block in enterocytes”

Model parameters

S1 Table – Full set of model parameters, their values and provenance. Parameters marked with with “**PE**” as provenance indicate that their values were estimated by nonlinear regression based on the data of the calibration experiments [13, 15, 27, 28]. Where a reference exists in brackets next to **PE**, it means that the value was initialized to the published value but allowed to be adjusted from there in the nonlinear regression. Whenever only references are indicated as provenance, then the value was used directly as in the literature without any adjustments.

Reaction	Reaction equation	Rate law	Parameter	Value	Provenance
**DMT1 endocytosis free**	$DMT1_{active} \rightarrow DMT1_{cytoplasm}$	$k_{cat} \times DMT1_{active}$	k_{cat}	29.4 s^{-1}	**PE**
**DMT1 endocytosis LIP modified**	$DMT1_{active} \rightarrow DMT1_{cytoplasm}$	$k_{cat} \times DMT1_{active} \times \frac{LIP^n}{K_m^n + LIP^n}$	k_{cat}	14.5 s^{-1}	**PE**
			K_m	2.805 M	**PE**
			n	1.03	**PE**
**DMT1 fusion**	$DMT1_{cytoplasm} \rightarrow DMT1_{active}$	$k_{cat} \times DMT1_{cytoplasm}$	k_{cat}	50 s^{-1}	**PE**
**DMT1 iron transport**	$Fe_{upper} \rightarrow LIP$	$k_{cat} \times DMT1_{active} \times \frac{Fe_{lumen}}{K_m + Fe_{lumen}}$	k_{cat}	6844 s^{-1}	**PE** ([1])
			K_m	2.835 M	[1]
**FT Fe Oxidation**	$2\,LIP \rightarrow DFP$	$\frac{k_{cat} \times \frac{H + rO}{24 + rO} \times FT_{cage} \times LIP^n}{Km^n + LIP^n}$	k_{cat}	591 s^{-1}	[2]
			K_m	0.35 mM	[3]
			n	1.3	[3]
			rO	2	[4]
			H	24	[4]
**FT Fe Reduction**	$DFP \rightarrow 2\,LIP$	$k_{deg} \times DFP$	k_{deg}	0.2605 s^{-1}	[2,3]
**FT Nucleation**	$2\,DFP \rightarrow 4\,core$	$k_{cat} \times DFP^2 \times FT_{cage} \times \frac{L + rN}{24 + rN} \times \frac{Ki^n}{Ki^n + core^n}$	k_{cat}	5x10^{7} s^{-1}	[4]
			K_i	0.4615 mM	
			n	4	
			rN	50	
			L	0	
**FT Core Formation**	$DFP \rightarrow 2\,core$	$\frac{k_{cat} \times DFP \times core}{Km + DFP} \times \frac{Ki^n}{Ki^n + core^n}$	k_{cat}	0.101564 s^{-1}	[4]
			K_m	5x10^{-6} M	
			K_i	4.6458 mM	

			n	4	
			m	8	
**FT core release**	$core \rightarrow LIP$	$k_{deg} \times \left(\frac{core}{FT}\right) \times FT_{cage}$	k_{deg}	5.46 μs^{-1}	[5]
**FT degradation**	$FT_{cage} \rightarrow$	$k_{deg} \times FT_{cage}$	k_{deg}	5.46 μs^{-1}	**PE** ([5])
**FPN-inactivation**	$FPN_{active} \rightarrow FPN_{inactive}$	$k_{cat} \times FPN_{active} \times \frac{Fe_{blood}^{n}}{K_m^n + Fe_{blood}^{n}}$	k_{cat}	1.44 μs^{-1}	[6–8]
			K_m	12.21 μM	[9]
			n	2.72	**PE**
**FPN-activation**	$FPN_{inactive} \rightarrow FPN_{active}$	$k_{cat} \times FPN_{inactive}$	k_{cat}	0.437 ps^{-1}	[A5]
**FPN iron transport**	$LIP \rightarrow Fe_{blood}$	$k_{cat} \times FPN_{active} \times \frac{LIP}{K_m + LIP}$	k_{cat}	1.88 s^{-1}	**PE** ([7, 10])
			K_m	2.31 μM	**PE** ([9, 10])
**IRPs inactivation**	$IRPs_{active} \rightarrow IRPs_{inactive}$	$k_{cat} \times IRPs_{active} \times LIP$	k_{cat}	4 M^{-1}s^{-1}	[11, 12]
**IRPs activation**	$IRPs_{inactive} \rightarrow IRPs_{active}$	$k_{cat} \times IRPs_{inactive}$	k_{cat}	4.63 μs^{-1}	
**FT expression**	$\rightarrow FT_{cage}$	$k_{cat} \times \left(1 - \frac{IRPs_{active}^{n}}{K_m^n + IRPs_{active}^{n}}\right)$	k_{cat}	0.0768 ps^{-1}	**PE** ([13,14])
			K_m	14 pM	[13]
			n	1	**PE**
**Body Sequestration**	$Fe_{Blood} \rightarrow Fe_{body}$	$k_{cat} \times Fe_{Blood}$	k_{cat}	0.000329 s^{-1}	[15, 16]
**Basal Uptake**	$Fe_{Blood} \rightarrow LIP$	$k_{cat} \times Fe_{Blood}$	k_{cat}	2.22 x 10^{-16} s^{-1}	[15, 16]
**Paracellular Iron Movement**	$Fe_{upper} \leftrightarrow Fe_{Blood}$	$k_{for} \times Fe_{upper} - k_{rev} \times Fe_{Blood}$	k_{for}	3.88 x 10^{-22} s^{-1}	[13]
			k_{rev}	3.17 x 10^{-15} s^{-1}	

Compartment	Connections	Notes	Unit	Value	Provenance
**Cell**	See Fig 1 for organization of compartments	-	volume	1.4 x 10^{-12} L	[17–19]
**Apical Membrane**		-	area	1.5 x 10^{-5} cm^{2}	[20, 21]
**Upper (Lumen)**		The lumen and blood compartment volumes are set by the experimental context. Thus these compartments sizes are determined based on the corresponding experimental set up	volume	6.67 x 10^{-10} L	-
**Lower (Blood)**			volume	8.57 x 10^{-10} L	-
**Basolateral Membrane (BLM)**		-	area	1.5 x 10^{-7} cm^{2}	[20–22]

Initial values	Biologic equivalent	Value Determination	Value	Provenance
**FT cage**	Intracellular FT cage polymers	Set by steady state simulations with initial values informed by references and parameter estimation	2.37519 x 10^{-9} M	[5, 23, 24]
**Core**	Oxidized mineral core iron in all FT cages		3.68222 x 10^{-6} M	[4, 25]
**DFP**	Oxidized non mineral core iron in all FT cages		1.34477 x 10^{-10} M	[4]
**LIP**	Labile iron pool (amount of labile cytoplasmic iron)		1.22388 x 10^{-7} M	[23, 26]

**DMT1 – active**	Active DMT1 on apical membrane capable of transporting luminal iron to cytoplasm	Set by steady state simulations with initial values informed by references and parameter estimation constrained to ratio in [19]	3.12308×10^{12} mol cm^{-2}	[27–29]
**DMT1 – vesicular**	DMT1 endocytosed to vesicles off of the apical membrane, does not transport luminal iron		1.87696×10^{-12} mol cm^{-2}	
**Fe_blood**	Iron found in blood compartment	Set by steady state simulations with initial values informed by references and parameter estimation	4.95846×10^{-9} M	[15, 16]
**Fe_lumen**	Iron found in apical lumen compartment	Set before simulation, aka iron dose	1.25×10^{-8} M	-
**FPN – active**	Active FPN on basal membrane capable of transporting cytoplasm iron to blood	Set by steady state simulations with initial values informed by references and parameter estimation. Note that these are relative not absolute values.	9.97975×10^{-14} mol cm^{-2}	[6, 7,10]
**FPN – internalized**	FPN endocytosed to vesicles off of the basal membrane, does not transport iron		2.02504×10^{-16} mol cm^{-2}	
**IRPs – active**	IRP1/2 combined species that can either inhibit FT production or is inactivated by LIP iron	Set by steady state simulations with initial values informed by references and parameter estimation. Note that these are relative not absolute values	6.88934×10^{-11} M	[12, 14, 30]
**IRPs – inactive**			7.26435×10^{-12} M	
**apc**	Atoms of iron per FT cage	The initial average value of iron atoms per FT cage, this value is estimated per experiment, as in each experiment the initial state may be different	1141.44 ; 3000	**PE**
**Body_fe**	Iron outside of blood compartment	Iron sink for simulation and output tracking	0 M	-
**Parameters for simulation control**	**Biologic meaning**	**Additional notes**	**Value**	**Provenance**
**Dose-fe**	Test dose of iron	Applied at time = time_delay, during simulation	0 – 125 nM	**PE** ([31, 32])
**Dose-0**	Blocking dose of iron	Applied at time 0 of simulation		
**Time_delay**	Time interval between doses of iron	Set for each simulation run, x-axis value of Fig 3A-3D and 4A-4H	0 – 72 hrs	[31]
**apc**	Atoms of iron per FT cage	Represents the average number of iron atoms per FT cage, calculated in real time; the range allowed for this value was obtained from the references	0 – 4300 atoms	[2–4]

References

1. Gunshin H, Mackenzie B, Berger UV, Gunshin Y, Romero MF, Boron WF, et al. Cloning and characterization of a mammalian proton-coupled metal-ion transporter. Nature. 1997;388: 482–488. doi:10.1038/41343

2. Sun S, Chasteen ND. Ferroxidase kinetics of horse spleen apoferritin. Journal of Biological Chemistry. 1992;267: 25160–25166. doi:10.1016/S0021-9258(19)74019-8

3. Tosha T, Hasan MR, Theil EC. The ferritin Fe2 site at the diiron catalytic center controls the reaction with O2 in the rapid mineralization pathway. Proc Natl Acad Sci U S A. 2008;105: 18182–18187. doi:10.1073/pnas.0805083105

4. Masison J, Mendes P. Modeling the iron storage protein ferritin reveals how residual ferrihydrite iron determines initial ferritin iron sequestration kinetics. PLoS One. 2023;18: e0281401. doi:10.1371/journal.pone.0281401

5. Mitchell S, Mendes P. A computational model of liver iron metabolism. PLoS Comput Biol. 2013;9: e1003299. doi:10.1371/journal.pcbi.1003299

6. Halleux C, Schneider Y-J. Iron absorption by CaCo 2 cells cultivated in serum-free medium as in vitro model of the human intestinal epithelial barrier. Journal of Cellular Physiology. 1994;158: 17–28. doi:https://doi.org/10.1002/jcp.1041580104

7. Kondaiah P, Sharp PA, Pullakhandam R. Zinc induces iron egress from intestinal Caco-2 cells via induction of Hephaestin: A role for PI3K in intestinal iron absorption. Biochem Biophys Res Commun. 2020;523: 987–992. doi:10.1016/j.bbrc.2020.01.023

8. Ranganathan PN, Lu Y, Fuqua BK, Collins JF. Discovery of a cytosolic/soluble ferroxidase in rodent enterocytes. Proc Natl Acad Sci U S A. 2012;109: 3564–3569. doi:10.1073/pnas.1120833109

9. Li S, Yang Y, Li W. Human ferroportin mediates proton-coupled active transport of iron. Blood Adv. 2020;4: 4758–4768. doi:10.1182/bloodadvances.2020001864

10. Eady JJ, Wormstone YM, Heaton SJ, Hilhorst B, Elliott RM. Differential effects of basolateral and apical iron supply on iron transport in Caco-2 cells. Genes Nutr. 2015;10: 14. doi:10.1007/s12263-015-0463-5

11. Ma J, Haldar S, Khan MA, Sharma SD, Merrick WC, Theil EC, et al. Fe2+ binds iron responsive element-RNA, selectively changing protein-binding affinities and regulating mRNA repression and activation. Proc Natl Acad Sci USA. 2012;109: 8417–8422. doi:10.1073/pnas.1120045109

12. Guo B, Phillips JD, Yu Y, Leibold EA. Iron regulates the intracellular degradation of iron regulatory protein 2 by the proteasome. J Biol Chem. 1995;270: 21645–21651. doi:10.1074/jbc.270.37.21645

13. Johnson G, Jacobs P, Purves LR. Iron binding proteins of iron-absorbing rat intestinal mucosa. J Clin Invest. 1983;71: 1467–1476.

14. Goforth JB, Anderson SA, Nizzi CP, Eisenstein RS. Multiple determinants within iron-responsive elements dictate iron regulatory protein binding and regulatory hierarchy. RNA. 2010;16: 154–169. doi:10.1261/rna.1857210

15. Parmar JH, Davis G, Shevchuk H, Mendes P. Modeling the dynamics of mouse iron body distribution: hepcidin is necessary but not sufficient. BMC Syst Biol. 2017;11: 57. doi:10.1186/s12918-017-0431-3

16. Parmar J, Mendes P. A computational model to understand mouse iron physiology and disease. PLOS Computational Biology. 2019;15. doi:10.1371/journal.pcbi.1006680

17. Buschmann RJ, Manke DJ. Morphometric analysis of the membranes and organelles of small intestinal enterocytes. I. Fasted hamster. J Ultrastruct Res. 1981;76: 1–14. doi:10.1016/s0022-5320(81)80046-9

18. MacLeod RJ, Hamilton JR, Bateman A, Belcourt D, Hu J, Bennett HP, et al. Corticostatic peptides cause nifedipine-sensitive volume reduction in jejunal villus enterocytes. Proc Natl Acad Sci U S A. 1991;88: 552–556.

19. Crowe PT, Marsh MN. Morphometric analysis of small intestinal mucosa. IV. Determining cell volumes. Virchows Arch A Pathol Anat Histopathol. 1993;422: 459–466. doi:10.1007/BF01606454

20. Mooseker MS, Tilney LG. Organization of an actin filament-membrane complex. Filament polarity and membrane attachment in the microvilli of intestinal epithelial cells. J Cell Biol. 1975;67: 725–743.

21. McConnell RE, Higginbotham JN, Shifrin DA, Tabb DL, Coffey RJ, Tyska MJ. The enterocyte microvillus is a vesicle-generating organelle. J Cell Biol. 2009;185: 1285–1298. doi:10.1083/jcb.200902147

22. Brown JW, McKnight CJ. Molecular Model of the Microvillar Cytoskeleton and Organization of the Brush Border. Schnur JM, editor. PLoS ONE. 2010;5: e9406. doi:10.1371/journal.pone.0009406

23. Alvarez-Hernandez X, Nichols GM, Glass J. Caco-2 cell line: a system for studying intestinal iron transport across epithelial cell monolayers. Biochimica et Biophysica Acta 1991;1070: 205–208. doi:10.1016/0005-2736(91)90165-5

24. Scheers NM, Almgren AB, Sandberg A-S. Proposing a Caco-2/HepG2 cell model for in vitro iron absorption studies. J Nutr Biochem. 2014;25: 710–715. doi:10.1016/j.jnutbio.2014.02.013

25. Salgado JC, Olivera-Nappa A, Gerdtzen ZP, Tapia V, Theil EC, Conca C, et al. Mathematical modeling of the dynamic storage of iron in ferritin. BMC Systems Biology. 2010;4: 147. doi:10.1186/1752-0509-4-147

26. Konijn AM, Glickstein H, Vaisman B, Meyron-Holtz EG, Slotki IN, Cabantchik ZI. The cellular labile iron pool and intracellular ferritin in K562 cells. Blood. 1999;94: 2128–2134.

27. Sharp P, Tandy S, Yamaji S, Tennant J, Williams M, Singh Srai SK. Rapid regulation of divalent metal transporter (DMT1) protein but not mRNA expression by non-haem iron in human intestinal Caco-2 cells. FEBS Lett. 2002;510: 71–76. doi:10.1016/s0014-5793(01)03225-2

28. Tandy S, Williams M, Leggett A, Lopez-Jimenez M, Dedes M, Ramesh B, et al. Nramp2 Expression Is Associated with pH-dependent Iron Uptake across the Apical Membrane of Human Intestinal Caco-2 Cells. Journal of Biological Chemistry. 2000;275: 1023–1029. doi:10.1074/jbc.275.2.1023

29. Hubert N, Hentze MW. Previously uncharacterized isoforms of divalent metal transporter (DMT)-1: Implications for regulation and cellular function. Proc Natl Acad Sci USA. 2002;99: 12345–12350. doi:10.1073/pnas.192423399

30. Galy B, Ferring-Appel D, Becker C, Gretz N, Gröne H-J, Schümann K, et al. Iron Regulatory Proteins Control a Mucosal Block to Intestinal Iron Absorption. Cell Reports. 2013;3: 844–857. doi:10.1016/j.celrep.2013.02.026

31. Frazer DM. A rapid decrease in the expression of DMT1 and Dcytb but not Ireg1 or hephaestin explains the mucosal block phenomenon of iron absorption. Gut. 2003;52: 340–346. doi:10.1136/gut.52.3.340

32. McConnell EL, Basit AW, Murdan S. Measurements of rat and mouse gastrointestinal pH, fluid and lymphoid tissue, and implications for in-vivo experiments. J Pharm Pharmacol. 2008;60: 63–70. doi:10.1211/jpp.60.1.0008
