# Supplementary material for: Mathematical modeling reveals ferritin as the strongest cellular driver of dietary iron transfer block in enterocytes
Source: PLoS Comput Biol. 2025 Mar 7;21(3):e1012374. doi: 10.1371/journal.pcbi.1012374 (PMC11918390; doi:10.1371/journal.pcbi.1012374)
Supplement: S3 Text — (PDF) [file pcbi.1012374.s003.pdf]

Supplement file S3 Text

Masison & Mendes (2025) “Mathematical modeling reveals ferritin as the strongest cellular driver of dietary iron transfer block in enterocytes”

Analysis of blocking magnitude dependence on iron blocking dose

To further characterize the ability of the model to produce iron metabolic behavior consistent with *in vivo* studies, the model simulation output was compared to experiments from Frazer et al. 2003 [15].

Here the experimental data comes from Fig 6 of reference [15] (herefter “*Frazer figure”*), where the time interval between iron doses is held at 6 hrs, and the magnitude of the iron blocking dose varied between 0.5 mg and 20 mg. The data presented in the Frazer figure represent a small subsection of the analysis performed to produce Fig 3A of this manuscript. The domain of the Frazer figure corresponds to the data within the dashed red line shown overlayed onto Fig 3A.

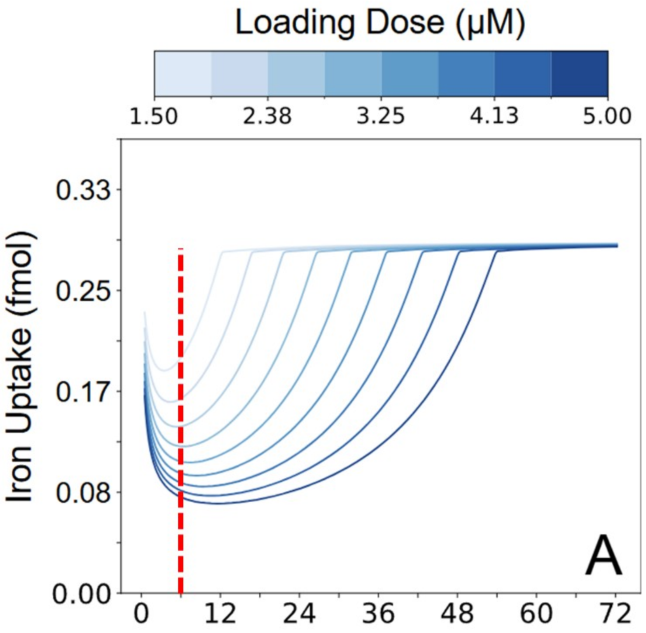

S1 Fig – Figure 3A of main manuscript annotated with the domain of the experimental results of the Frazer Figure. The blocking dose variation in the Frazer figure occurs at a constant time between (6 hrs), corresponding to the simulation data marked by the red dashed line.

Due to the differences in the Frazer experimental system vs our model (whole animal vs single cell), the doses used to produce the Frazer figure (0.5 – 20 mg) were scaled appropriately to a concentration (0.125 – 5 μM). Additionally we have to change our model’s initial conditions to match those of the Frazer figure (namely, initial blocking dose set to match their varied dose, and time interval between doses set to 6 hrs). After these changes, we ran simulations with our model so the data could be plotted together for comparison. The results are shown in S2 Fig.

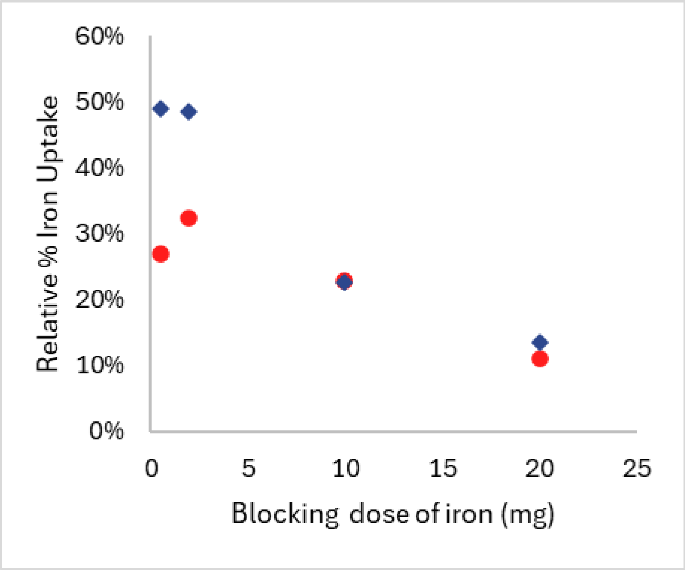

S2 Fig – Effect of blocking iron dose on the relative amount of iron uptake. In both panels the x-axis shows the iron blocking dose (mg) and the y-axis shows the relative change (%) of iron uptake relative to the test dose (100% would mean no block, 0% would be a complete block). The Figure shows the result of using the 4 blocking doses of iron, as used in Frazer (red circles indicate the data from the Frazer figure, blue diamonds indicate data from our model).

These results show further validation that the present model behaves in accordance with *in vivo* experimental observations, especially within the doses used in model simulation for the rest of the manuscript (namely 10-20 mg).
