# Supplementary material for: Mathematical modeling reveals ferritin as the strongest cellular driver of dietary iron transfer block in enterocytes
Source: PLoS Comput Biol. 2025 Mar 7;21(3):e1012374. doi: 10.1371/journal.pcbi.1012374 (PMC11918390; doi:10.1371/journal.pcbi.1012374)
Supplement: Attachment — Submitted filename: Mendes review fin2.pdf [file pcbi.1012374.s005.pdf]

Review of Masison and Mendes… Mathematical modeling reveals…

In this study, the authors mathematically modeled "mucosal iron block" in which intestinal cells (enterocytes) in the duodenum resist importing iron some hours after being exposed to an initial dose of iron. There are a number of influences involved, including DMT1, an iron importer, ferritin (FT), an iron-storage protein, and IRP proteins that regulate FT expression. All of these are sensed by the labile iron pool (LIP). The iron that enters the cell through DMT1 becomes part of the LIP. The LIP binds TF and the IRP proteins reversibly. All of these processes are occurring simultaneously to achieve a steady-state. The objective of their study was to evaluate which factors control (or drive) mucosal iron block. The study is overall reasonable, and it appears that they have made significant advances in explaining the biochemistry underlying this phenomenon. Thus, the manuscript is potentially an important contribution to the mathematical model of iron metabolism in cells and the human body. However, model development needs to be described better. The PI is a well-known and respected expert in this area who has a reputation for designing models carefully and with strong documented connections to the biology and biochemistry, so the issues of concern may simply be those of presentation style – I'm not sure. However, the concerns mentioned below need to be addressed before the manuscript can be further evaluated.

Comment 1: I was unable to open the SI files, so I apologize if the requested information is contained there. It would be helpful to readers generally if SI files were in Word or pdf format. The model involves five cellular compartments including the intestinal lumen, apical membrane of enterocytes, the cytosol of these cells, the basolateral membrane of these cells, and blood plasma, but the compartments were insufficiently described. Does each have a specific volume? If the volumes are different, the rates of a species leaving one region and entering another will be unequal, assuming rates are defined as concentration change per time change. How was this treated in the model?

Comment 2: The model includes many (68+19 = 87!) adjustable parameters, which is large for a model that only has 19 reactions. They should be listed and some indication how the values for each was assigned should be included. I've found that in many cases, data taken from the literature isn't fully compatible with the model assumptions. These incompatibilities ought to be discussed in the table, including how they were resolved. If a parameter was assigned by nonlinear least-squared fitting (line 144), the assumed fitting function ought to be indicated as well.

Comment 3: Along these lines I appreciated the list of reactions, rate-law expressions, and parameters given in Tables 3-6. Since there are only 3 more reactions in the model, they should be included in a final table for completeness. I noticed that the K_M for the LIP in the DMT1 endocytosis rate (Table 3) was 2.805 M – MOLAR! Ditto for the K_M of Fe in the lumen – 2.835 M! I've never seen values so high. Since the LIP and Fe-lumen concentrations are nM – uM, the Michael-Menten structure for these rate laws seems inappropriate as those values of K_m indicate first-order in these substrates with an effective rate-constant k_{cat}/K_M. How were these Km values assigned?

Comment 4: Some parameters (e.g. H rO, L, core, apc) seemed to have not been defined but should be. Regarding the stoichiometry of the reaction in which LIP binds FT, it seems from the

reactions of Table 4 that the number is far less than 4300. If each LIP has 1 iron, then DFP has 2 Fe and core has 1 iron. Is only 1 iron presumed to bind apo ferritin?? This needs to be explained.

Comment 5: The modeling of FT seems unnecessarily complicated given their objective to make the model "minimal" (line 125). Why include DFP, core and cage? Why not just have 4300 LIP molecules bind FT reversibly? I suspect this complexity is a carry-over from an earlier modeling study of FT.

Comment 6: Figure 1 was helpful but the symbols need to be better explained. There are boxes vs circles, circles with diagonal lines through them, and lines with or without arrows; all of these need to be explained. Does the size or color of the circle matter? Does it matter whether a circle or rounded box is used? Why are there boxes contained along a line?

Comment 7: The functions used to fit the data of Figure 2 needs to be described. In C, the function seems to be an inverted parabola but the data would seem to prefer a hyperbolic function. The function used in plot E seems to show a discontinuity at 24 hours. Is one function assumed for 0 – 24 hr and a different function for 24 – 72 hrs? There is a similar though less dramatic issue with plot F. In Fig 3 left top, the curve for iron uptake showing mucosal block seems to have a discontinuity at 36 hours. Same for the simulations in A and B; why? Why shouldn't the ODE system yield smoothly changing plots?

Comment 8: I'm confused by the units that are used in the plots. Many plots have various types of "amounts" of a component; e.g. pmoles, particles, or "iron transfer amount". On the other hand, Figure 2B uses the concentration of DMT1 (in picomolar), and Table 1 includes concentrations for the model components. If the ODE system uses concentrations rather than moles, as is typical, why are plots sometimes given in "amounts" like pmoles or "particles'?

Comment 9: I would simplify and consolidate the plots in Figures 3 - 5, and label them better. In general, there are too many plots and insufficiently simple explanations of what they show. In Figure 3, E and G might be consolidated into a single plot by using colored lines. Same for plots F and H. The abscissas for plots A, B, C, and D are identical and the ordinates are all iron amounts; perhaps they could be consolidated into a single panel. The ordinate legends are also insufficiently titled in terms of explaining what exactly is being plotted. In Figure 4, the axes indicate "Particles" or "Time" or "relative value". A more descriptive title would be helpful; e.g. "picomoles of total iron uptaken into the cell" instead of "Particles". Same issue for defining the ambiguous terms "Time" and "relative value" – give more details to help the reader understand the plot. Figure 5 could be similarly simplified, consolidated and described better. Full plots could be included in the SI.

Comment 10: The authors argue in the discussion that "a major utility of computational models" is to isolate and modify a particular system component, while keeping the rest of the system unaltered. I can see their point in comparison with in-vivo experiments; a computational system can be manipulated to isolate particular aspects of a larger system. However, the argument also seems opposite of what I regard as a major advantage of ODE-based computational models – namely their ability to ***integrate*** numerous disparate influences to see how the unified system behaves when all these influences are active. Why minimize this? The authors also downplay the

ability of in vitro experiments to isolate and study a particular factor; this is part of the reductionistic agenda of biochemistry.

Comment 11: They also seem to downplay the central role of the LIP in integrating these factors. The endocytosis of DMT1 depends on LIP. The extent of FT sequestration depends on LIP. The regulation of FT depends on IRPs, which depends on blood Fe which depends on LIP and FPN. All effects involve the LIP and all are integrated by the model to generate the system. Why downplay this?

Comment 12: Line 29: The authors state: "Analysis reveals that ferritin and iron regulatory protein regulation are the main drivers of mucosal block, secondary to dose magnitude and regulation by hepcidin." How can they be the main drivers if they are secondary to dose magnitude and regulation?

Comment 13: Line 373: they should use an equation editor to present those equations. Also, I don't think they define R…

Comment 14: line 390: Table 2 should include units for each entry.

Comment 15: Line 71: "there has been a distinction between the mucosal iron absorption block mediated by hepcidin and a local intestinal block that is dependent on the biochemistry of individual enterocytes…In this work, we use computational modeling to estimate the quantitative impact of distinct cellular mechanisms within individual enterocytes …" This sounds like the effects of hepcidin are not considered in their model, but in the rate-law for FPN inactivation, the Fe concentration in the blood is a factor, which suggests that hepcidin is indirectly being considered. This should be clarified.

Comment 16: I believe that iron-loaded enterocytes (when FPN has been inactivated) are sluffed off the intestinal lining and exit the body, and that this prevents iron absorption. Shouldn't this relevant process be included in their model?

Comment 17: Line 274: …"the more sensitive the degradation of FPN is to the level of hepcidin, the smaller the amount of iron transferred to the plasma is". I would think that the more sensitive the process is to hepcidin, the more ***sensitive*** the cell controls the export of iron to the plasma.

Comment 18: Do mammalian cells in culture (e.g. Caco-2 cells) exhibit the mucosal block phenomenon? Or is this something only observed in the intestines of mammals? The answer will impact the utility of using literature results involving treatments of mammalian cells in culture with iron.

Summary: Despite the many concerns just listed, the modeling study overall was carefully designed and connected to the known biology and biochemistry. The analysis seems appropriate. Model development was (pretty) well explained, though there need to be some improvements as indicated above. In summary, I recommend that the authors be offered the opportunity to respond to these comments and revise the manuscript appropriately.
