# Supplementary material for: Mathematical modeling reveals ferritin as the strongest cellular driver of dietary iron transfer block in enterocytes
Source: PLoS Comput Biol. 2025 Mar 7;21(3):e1012374. doi: 10.1371/journal.pcbi.1012374 (PMC11918390; doi:10.1371/journal.pcbi.1012374)
Supplement: Attachment — Submitted filename: ResponseReview.pdf [file pcbi.1012374.s007.pdf]

We would like to thank all three reviewers and the editors for giving us the opportunity to improve our manuscript following their numerous comments. We hope that we were able to respond positively to most, if not all, of these such that the manuscript may now be considered acceptable to PLOS Computational Biology.

In this document we indicate the reviewer's questions in black sans serif font and our replies in blue roman font.

Reviewer #1

An interesting paper that uses mathematical modeling in an effort to define duodenal intestinal iron uptake and the mucosal block in enterocytes. The authors have an in depth understanding of intestinal iron metabolism and provide reasonable rationale for the simplification of the processes involved in iron transport.

Thank you for your assessment, it is indeed our intention to use computational means to better understand the biological system.

The authors may want to consider a short paragraph – perhaps in the results – where they summarize the assumptions and refer the readers to the methods for further information. Some of the assumptions, such as Hephaestin not contributing to mucosal block, may not be entirely accurate as a key finding in mice mutant in this gene is enterocyte accumulation of iron in ferritin to very high levels – a constitutive mucosal block. However, as this represents a non-physiologic state, it is reasonable to exclude from the model as they do.

We agree that it is important to be clear about assumptions of a model, as they need to be considered when assessing the model results. Although we already dedicate a long section of text on assumptions of the model (at the end of Methods), we now added, as suggested, a short paragraph in the Results section bringing attention to a list of assumptions:

"All models by necessity carry assumptions and simplifications, and that is true in the present model. Specifically, 1) when protein synthesis is included this is represented by only one reaction producing the mature protein, 2) heme iron uptake is not considered in this model, 3) the role of the ferroxidases DCYTB and HEPH are not included, 4) only ferritin is post-translationally regulated by IRPs, 5) the distinct action of IRP1 and IRP2 are aggregated into a single variable "IRPs". Further details about these assumptions can be found in Methods."

Regarding the Hephaestin results mentioned by the reviewer, we now describe them in the respective section on the Hephaestin assumption; we added:

"While a mutation in hephaestin is known to result in accumulation of non-heme iron in enterocytes and in a reduced transfer of iron to plasma [102], it appears that it is responsive to global levels of iron rather than the level of iron in the enterocyte [103], and as such we assume that it does not have a role in the mucosal block caused by consecutive dietary iron doses. The pathological iron mucosal block caused by the HEPH mutation is not addressed in this model."

and accordingly added two references:

102. Vulpe CD, Kuo YM, Murphy TL, Cowley L, Askwith C, Libina N, et al. Hephaestin, a ceruloplasmin homologue implicated in intestinal iron transport, is defective in the sla mouse. Nat Genet. 1999;21: 195–199. doi:10.1038/5979

103. Chen H, Su T, Attieh ZK, Fox TC, McKie AT, Anderson GJ, et al. Systemic regulation of Hephaestin and Ireg1 revealed in studies of genetic and nutritional iron deficiency. Blood. 2003;102: 1893–1899. doi:10.1182/blood-2003-02-0347

One possible contribution to the regulation of intestinal iron uptake that the authors do not explicitly discuss is the role of enterocyte sloughing and turnover which has been suggested by some authors to play a role in preventing the iron bound in ferritin as a result of the mucosal block in ever being absorbed.
It would be interesting to consider the kinetics of their model with the kinetics of intestinal cell turnover – especially in the 48-72 hour period after a mucosal blocking dose when new enterocytes will likely constitute a significant portion of the transporting enterocytes and similarly a portion of the iron bound to ferritin in the enterocytes will be lost by turnover. This reviewer wonders whether taking this turnover into account would improve the model. Is the iron in Ferritin in the enterocyte – a dead end – in that it is unlikely to be mobilized within the time scale of the enterocyte lifespan or alternatively could it serve to buffer iron uptake as they suggest for DMT1 to prevent excess iron uptake.

We share this question as well, and in fact we have planned a further detailed study that would include the tissue dynamics. Our approach is a bottom-up strategy, where we started with the lower molecular principles and build up to the tissue and organism level. However, to include the tissue level requires building a multiscale model – where the cellular dynamics will be represented by an agent-based model, while the intracellular dynamics by this ODE model. We have started working towards that approach, but that is a complex piece of modeling and so that work is far from complete (incidentally the first author of this manuscript is now starting residency after completion of his MD/PhD degree and won't have time to be the main researcher on that project). That complex computational project requires that we refine a few new techniques. Thus we feel that it needs to be the subject of a future follow-up research project and publication.

Our preliminary calculations, while we were drafting that project, indicate that indeed a lot of ferritin-bound iron is likely to end up being lost by sloughing, but this is currently too speculative and we prefer not to include this observations in the manuscript.

We added a paragraph to the discussion covering the sloughing process:

"The high buffering capacity of FT also has consequences at the tissue-level given that duodenal enterocytes have short life span and are sloughed to the intestinal lumen at a considerable rate [104]; thus it is expected that such sloughing of enterocytes with high ferritin-bound iron content will result in irreversible loss of iron that had been uptake by enterocytes but will be irreversibly lost from being transferred to the rest of the body. To investigate such dynamics, however, is beyond the scope of this work and will require a multiscale model of the villus structure and dynamics."

The authors also don't explicitly discuss the role of very high duodenal iron leading to NTBI in the enterohepatic circulation – clearly the mucosal block is not effective at some threshold and is there any data in the literature that could give some insight into this phenomenon, e.g. when does the model they provide breakdown - especially if a potential use of the model is to help develop/understand physiologic iron uptake or an overall model of iron homeostasis as a building block. It is important to understand the intestinal iron doses where the model is appropriate to utilize and when the model is likely to be violated.

This is a very good question and it would be a good test for the model. However we have not found any solid literature showing accumulation of NTBI derived solely from diet in non-hemochromatosis individuals. Of course, in hemochromatosis there are large levels of NTBI but this is derived from a reduced negative feedback loop through hepcidin. We could not find data on enterocyte iron transfer under large iron doses, thus any simulation could not currently be confirmed.

In Fig. 3 we attempted to show the effect of gradually increasing doses of iron on uptake, transfer, and blocking.

Fig. 3A shows that our model predicts longer and somewhat more pronounced blocking at higher dietary doses, this is better summarized in Fig. 3E that shows the dependence of the time interval at which there is maximal block on iron loading dose, which nearly doubles from 1.5 to 5.0 µM doses. The recovery time (illustrated in Fig. 3 legend as number 3, the time until the inflection point in the uptake curve) increases even more steeply. Fig. 3C shows that at higher doses (darker curves) the blocking is more pronounced and takes longer.

In order to partly answer your question, we now tried a loading dose of 10.0 µM, 2-fold larger than the highest in Fig 3C, the result is an even longer block; note that the minimum is not achieved even at 72 hour between doses. This seems to be derived from ferroportin becoming saturated for the entire interval between doses (at initial doses larger than about 3.6 µM). We were not able to find any experimental data that we could compare to this, so we opt to not discuss the 10.0 µM initial dose and leave Figs 3A and 3C going only up to 5 µM.

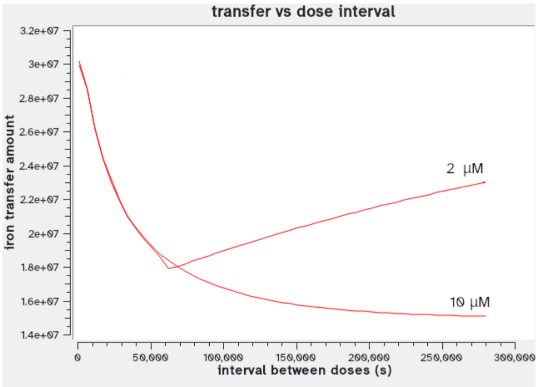

Reviewer #2

I was unable to open the SI files, so I apologize if the requested information is contained there. It would be helpful to readers generally if SI files were in Word or pdf format.

The SI files are COPASI files (extension .cps), data files (extension .tsv), and Excel files (extension .xlsx). We prefer to supply the raw data that allows reproducing our results. We understand that if you don't have COPASI installed, then it would be useful to display the equations in a PDF (as requested by Reviewer #3 as well). We have now added a PDF file with the equations of the mathematical model.

The model involves five cellular compartments including the intestinal lumen, apical membrane of enterocytes, the cytosol of these cells, the basolateral membrane of these cells, and blood plasma, but the compartments were insufficiently described. Does each have a specific volume? If the volumes are different, the rates of a species leaving one region and entering another will be unequal, assuming rates are defined as concentration change per time change. How was this treated in the model?

The cell, upper (lumen) and lower (blood) compartments are three-dimensional and all have different volumes, the apical membrane and basolateral membranes are two-dimensional compartments and have different areas. The table below (now included in table S1) shows their sizes:

Model compartment	Dimension	Size
Upper (intestinal lumen in the vicinity of the cell)	3D	6.67×10^{-10} l
Lower (blood volume in the vicinity of the cell)	3D	8.57×10^{-10} l
Cell (actually, cytoplasm of the cell)	3D	1.4×10^{-12} l
Apical membrane	2D	1.5×10^{-5} cm^2
Baso lateral membrane	2D	1.5×10^{-7} cm^2

COPASI makes all calculations in terms of numbers of molecules rather than concentration, so the different compartment sizes are automatically taken into consideration (essentially while the user specifies concentrations, internally they are converted to molecule numbers for all calculations). The rates of transport are thus in molecules per unit time and this is, of course, the same on both sides of the membrane irrespective of their volumes (they would be different in terms of concentration per unit time). This can be confirmed in the differential equations now provided in supplement.

The model includes many (68+19= 87!) adjustable parameters, which is large for a model that only has 19 reactions. They should be listed and some indication how the values for each was assigned should be included. I've found that in many cases, data taken from the literature isn't fully compatible with the model assumptions. These incompatibilities ought to be discussed in the table, including how they were resolved. If a parameter was assigned by nonlinear least-squared fitting (line 144), the assumed fitting function ought to be indicated as well.

We now included the requested table in the supplement S1 PDF file.

The 19 parameters mentioned (in 68+19) are only needed for simulation manipulations or measurements, and are set by us accordingly; they were not estimated nor do they affect the model itself. Thus there are only 68 true model parameters. Of these 55 were set by values obtained from the literature and only 13 were actually estimated from the calibration data.

The fitting function is a (long) sum of the square of differences between data and corresponding model simulation (also known as residuals), which is set up by COPASI automatically given the data supplied. Therefore this is a typical nonlinear least-squares problem. All the least-squares fits that were carried out are displayed in Figure 2A-C. Figure 2D-F depicts the model validation: in this case the parameter values obtained from fitting (Fig. 2A-C) were used to simulate the experiments (*i.e.* no fitting happened in panels D-F).

The assumptions taken in the model are discussed in methods. While the parameter values taken from the literature inevitably carry assumptions, we did not explicitly try to check their consistency, however the best test here is the validation simulations, which show that the model parameters are in good agreement with *in vivo* data. This validation is important because those are conditions not used in the fitting. Panels E and F cover an entirely different time scale than the experiments used for fitting, and this demonstrates the extrapolation power of the model (not perfect in the case of panel E, but rather good in panel F). If there were inconsistent underlying assumptions in the data used, they do not seem to be consequential.

Along these lines I appreciated the list of reactions, rate-law expressions, and parameters given in Tables 3-6. Since there are only 3 more reactions in the model, they should be included in a final table for completeness.

We have added an extra table displaying the remaining 3 reactions (as below):

Table 7. Additional reactions. Shown below are the additional three reactions in the model, their stoichiometries, rate laws, and rate laws parameter values. The reactions represent rest of body sequestration of plasma (blood) iron ("Body Sequestration"), basal membrane enterocyte iron uptake from plasma ("Basal Uptake"), and paracellular iron movement direct from lumen to plasma, reversibly ("Paracellular Iron Movement").

Reaction	Reaction	Rate Law	Paramet er	Value	Ref.
**Body Sequestration**	$Fe_{Blood} \rightarrow Fe_{body}$	$k_{cat} \times Fe_{Blood}$	k_{cat}	0.000329 s^{-1}	-
**Basal Uptake**	$Fe_{Blood} \rightarrow LIP$	$k_{cat} \times Fe_{Blood}$	k_{cat}	2.22 x 10^{-16} s^{-1}	-
**Paracellular Iron Movement**	$Fe_{upper} \leftrightarrow Fe_{Blood}$	$k_{for} \times Fe_{upper} - k_{rev} \times F$	k_{for}	3.88 x 10^{-22} s^{-1}	-
			k_{rev}	3.17 x 10^{-15} s^{-1}	-

I noticed that the KM for the LIP in the DMT1 endocytosis rate (Table 3) was 2.805 M – MOLAR! Ditto for the KM of Fe in the lumen – 2.835 M! I've never seen values so high. Since the LIP and Fe-lumen concentrations are nM – uM, the Michael-Menten structure for these rate laws seems inappropriate as those values of Km indicate first-order in these substrates with an effective rate-constant kcat/KM. How were these Km values assigned?

These large values of K_m indicate that the effect of the corresponding molecules on the rate of reaction are weak (but not zero). The K_m for lumen iron on DMT1 transport was obtained from reference 38 (Gunshin et al. 1997 Nature 388:482 doi:10.1038/41343) and so we did not fit that parameter to data and kept it at the value as reported.

Regarding the K_m for the activation effect of LIP on the rate of DMT1 endocytosis, this was indeed the result of the fit. To better reason about this value, we traced its profile likelihood in the global fit, as described by Kreutz et al. (2013 FEBS J. 280:2564–257 doi:10.1111/febs.12276) and Schaber (2012, BioSystems 110:183–185 doi:10.1016/j.biosystems.2012.09.003), and depicted in the plot below. Analysis of this profile confirms that: a) the value indicated (blue circle) is indeed the best solution, and b) larger values would cause a worse fit. The latter point is important as removing this effect from the model is equivalent to setting this parameter to infinity (and essentially remove the activation altogether), but this would result in a worse fit (see the right hand side of the profile, where the sum of squares of residuals increases, note this is a log-log plot). So, given this we would prefer to keep the hyperbolic activation of LIP on the DMT1 endocytosis in the model, even though recognizing that this effect is weak.

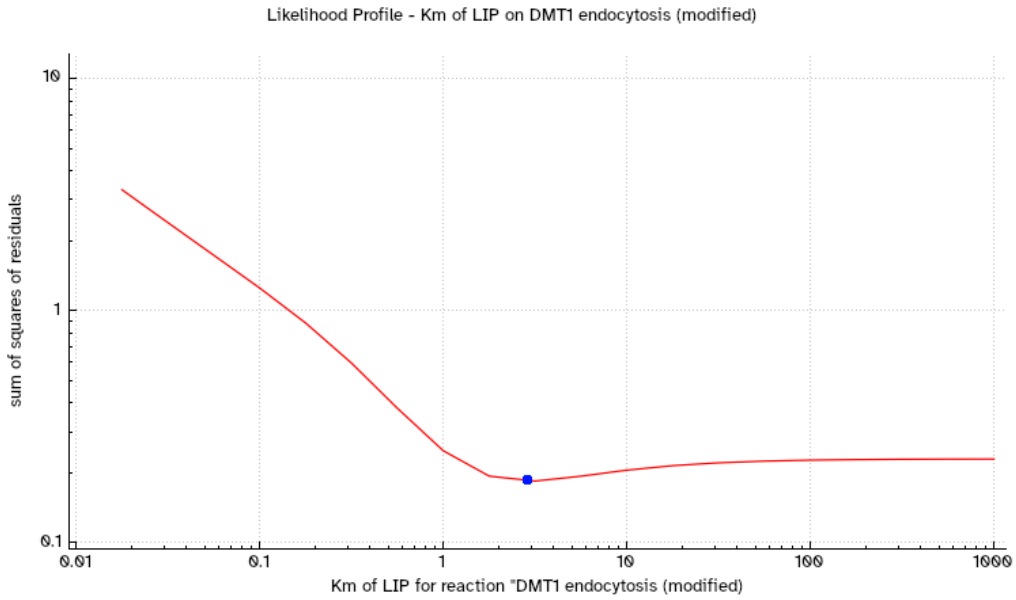

Some parameters (e.g. H rO, L, core, apc) seemed to have not been defined but should be. Regarding the stoichiometry of the reaction in which LIP binds FT, it seems from the reactions of Table 4 that the number is far less than 4300. If each LIP has 1 iron, then DFP has 2 Fe and core has 1 iron. Is only 1 iron presumed to bind apo ferritin?? This needs to be explained.

These variables are part of the Ferritin submodel which we had published in a previous peer reviewed publication and referenced in this manuscript (doi:10.1371/journal.pone.0281401, hereafter "ferritin paper"). While a minimal model, it is still sufficiently complex that it required a full publication to define and validate.

No, ferritin does not only have 1 iron bound (in fact the smallest number would be 2, since iron is added to the core as DFP and that has two iron atoms). The variable "core" is the equivalent concentration of iron that is trapped in the solid form if it were to be dissolved. To spare the reviewer having to read the entire ferritin paper, we describe these parameters and variables here:

- *H* and *L* characterize the (average) ferritin particles in this cell type. Ferritin is a 24-subunit polymer made of heavy ferritin subunits (FTH gene) and light ferritin subunits. Different tissues have different compositions of *H:L* (from 0:24 to 24:0); the rate of oxidation of LIP into DFP depends on this proportion (higher oxidation rate with higher proportion of H/L. H is an integer in the interval [0,24], L=24-H.
- *rO* is an empirical parameter that allows ferritin particles exclusively made of L subunits to still be able to catalyze the oxidation of LIP (free iron) to DFP; the parameter was set to 2 to match experimental data, as described in our ferritin publication,
- *core* is a variable that quantifies iron in the solid ferrihydrite particles inside ferritin; this variable is the concentration of iron that would be released if all ferrihydrite core particles were dissolved. We cannot say that it is the concentration of iron inside ferritin as this is a solid (and solids don't have concentration…), but it is the equivalent concentration if these particles were soluble (so if all were dissolved it would be the increase in LIP concentration)
- *apc* stands for "atoms per core" and it quantifies the average number of iron atoms in each core particle (*apc* can go from 0 to 4300)

The modeling of FT seems unnecessarily complicated given their objective to make the model "minimal" (line 125). Why include DFP, core and cage? Why not just have 4300 LIP molecules bind FT reversibly? I suspect this complexity is a carry-over from an earlier modeling study of FT.

The incorporation of iron into a solid core inside Ferritin particles is a complex process and is a nonlinear function of the LIP level (in fact a non-monotonic function). Ferritin particles can bind up to 4300 iron atoms (LIP particles) in a solid core, however the amount stored in each ferritin particle is variable. Modeling this is not trivial and our previous manuscript (the ferritin paper doi:10.1371/journal.pone.0281401) focused on developing a model to represent this complex process and yet be simple enough to be incorporated in larger (cellular) models. As mentioned above, while a minimal model, this is still more complex than could be reduced to one reaction. A simplistic one-reaction approach could not reproduce the experimental observations; for example the rate of incorporation of iron into ferritin increases with the amount of mineralized iron only up to a certain level (1500-2000 atoms) and then decreases (https://doi.org/10.1042/bj1430445). This is all described in our previous ferritin model paper and that's why we have re-included that model as a component of the larger enterocyte model here.

Figure 1 was helpful but the symbols need to be better explained. There are boxes vs circles, circles with diagonal lines through them, and lines with or without arrows; all of these need to be explained. Does the size or color of the circle matter? Does it matter whether a circle or rounded box is used? Why are there boxes contained along a line?

The reviewer's question is pertinent, in that often too many diagrams are used without standard meanings. In this case, however, the diagram in figure 1 was created with Cell Designer and used its convention, and this has been very widely used in systems biology publications. However, the reviewer's question parallel the motivation for the creation of the Systems Biology Graphical Notation standard (SBGN, see reference 100), that has since been adopted as a standard in systems biology. Thus, we modified the diagram to make it SBGN-compliant. The meaning of the symbols are defined in references 100 and 101 and we do not think appropriate to repeat the content of those publications in the figure legend (which is already long).

But, briefly, rounded rectangles represent macromolecules, ellipses represent other chemicals, circles with diagonal crosses indicate species that are not tracked in the model (*i.e.* boundaries of the model). Arrows with filled arrow heads and a square in the middle represent reactions (or transport), lines that go to the squares of reactions indicate: catalysis (circles), activation (empty arrow heads) or inhibition (blunt heads). Double-walled rectangles (in yellow) represent the membranes delineating compartments. The colors are not important but help distinguish the types of species involved.

The functions used to fit the data of Figure 2 needs to be described. In C, the function seems to be an inverted parabola but the data would seem to prefer a hyperbolic function. The function used in plot E seems to show a discontinuity at 24 hours. Is one function assumed for 0 – 24 hr and a different function for 24 – 72 hrs? There is a similar though less dramatic issue with plot F. In Fig 3 left top, the curve for iron uptake showing mucosal block seems to have a discontinuity at 36 hours. Same for the simulations in A and B; why? Why shouldn't the ODE system yield smoothly changing plots?

The fits in Figure 2 use the entire ODE model, thus its integral is obtained by numerical solution. As mentioned above, the fitting function is a sum of squares of residuals between the calibration experimental data and the corresponding model value (of course, obtained through numerical solution of the ODEs). The reason why the curves appear parabolic rather than hyperbolic is not easy to identify from the differential equations alone since we don't have an analytical solution (we're also not sure why to expect them to be hyperbolic). Also, note that 2E and 2F are not time courses; the abscissa is the interval of time between iron doses; for each data point in these plots, a whole time course is run until 30 min and then the amount of iron transferred/uptake is noted and

entered into these plots. So Figs. 2E and 2F were not properly labeled and we have updated the labels to "time between doses (h)" rather than simply "Time (h)".

Regarding the apparent discontinuities in Fig. 2E and Fig. 2F, when zooming in on the region of interest, we can show that the model does not actually have a discontinuity, it just changes direction very rapidly (the plots here are zoom-ins around the "kinks" and are in units of second rather than hours):

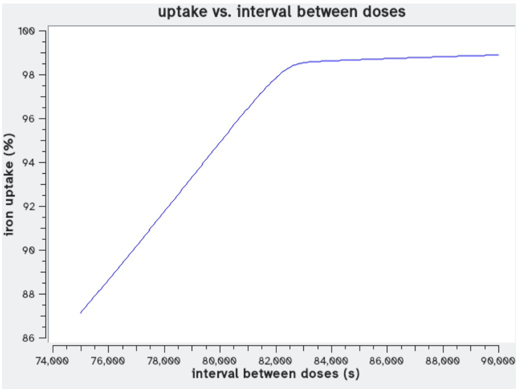

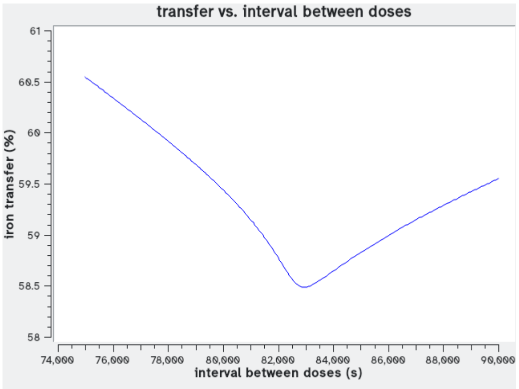

A similar thing is also happening in Figs. 3A-D (and the illustrative plots on the left). There is no discontinuity rather a very fast change that, in the scale used, appears as a discontinuity (here showing only one curve for each of Fig. 3A and Fig. 3C:

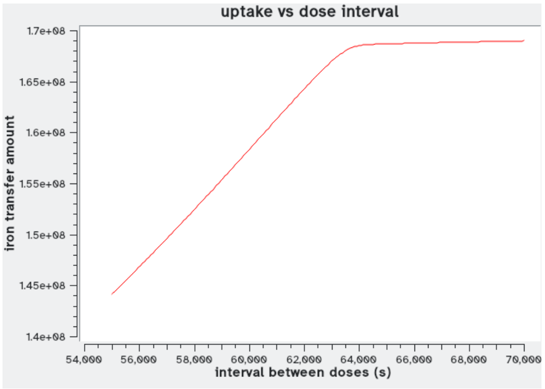

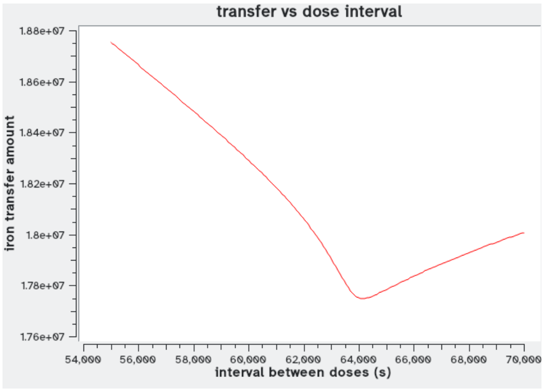

I'm confused by the units that are used in the plots. Many plots have various types of "amounts" of a component; e.g. pmoles, particles, or "iron transfer amount". On the other hand, Figure 2B uses the concentration of DMT1 (in picomolar), and Table 1 includes concentrations for the model components. If the ODE system uses concentrations rather than moles, as is typical, why are plots sometimes given in "amounts" like pmoles or "particles'?

As mentioned above, the ODEs are internally expressed in quantities rather than concentrations. When a species is restricted to a compartment it is easy to express it in concentration, but if it moves across compartments then it is not (which volume to use to calculate concentration?). So for DMT1 it is easy to express concentration, but the amount of iron transferred cannot easily be converted to a concentration – relative to what volume? The intestinal lumen? The plasma volume? The enterocyte volume? Whenever there is a flux between compartments of different volumes, fluxes have to be expressed in amount/time. The reviewer is right that it is better to express all quantities (amounts) in the same units, which we now do in fempto mole (fmol).

Incidentally, we also found a number of units that were incorrect in the manuscript and have now corrected them too.

I would simplify and consolidate the plots in Figures 3 - 5, and label them better. In general, there are too many plots and insufficiently simple explanations of what they show. In Figure 3, E and G might be consolidated into a single plot by using colored lines. Same for plots F and H. The abscissas for plots A, B, C, and D are identical and the ordinates are all iron amounts; perhaps they could be consolidated into a single panel. The ordinate legends are also insufficiently titled in terms of explaining what exactly is being plotted. In Figure 4, the axes indicate “Particles” or “Time” or “relative value”. A more descriptive title would be helpful; e.g. “picomoles of total iron uptaken into the cell” instead of “Particles”. Same issue for defining the ambiguous terms “Time” and “relative value” – give more details to help the reader understand the plot. Figure 5 could be similarly simplified, consolidated and described better. Full plots could be included in the SI.

These figures do indeed express rather complex phenomena and we tried to make them as simple as possible, but also displaying the complete behavior. We agree that some explanations in the legend are not sufficient and we have expanded them considerably, hoping that they now better explain the data. As for combining the plots, we disagree with the reviewer suggestion because, to us, that would actually make the plots more complicated to understand. For example Fig 3E and G display entirely different entities with different scales, to combine the plot would require different colors. We think that one can reduce the number of panels, *or* make the figures easier to understand – and these two criteria oppose each other. Respectfully we opt to keep the same number of panels.

The authors argue in the discussion that “a major utility of computational models” is to isolate and modify a particular system component, while keeping the rest of the system unaltered. I can see their point in comparison with in-vivo experiments; a computational system can be manipulated to isolate particular aspects of a larger system. However, the argument also seems opposite of what I regard as a major advantage of ODE-based computational models – namely their ability to integrate numerous disparate influences to see how the unified system behaves when all these influences are active. Why minimize this? The authors also downplay the ability of in vitro experiments to isolate and study a particular factor; this is part of the reductionistic agenda of biochemistry.

We share the same thoughts of the reviewer, however we do not see these two aspects of modeling being opposed. One of the great advantages of a model – and our main intention here – is to integrate several processes that experimentally have been studied in isolation. But another advantage of modeling is that it allows us to carry out experiments on specific components without other effects, which would not be easy to effect experimentally. Too better express this, we rewrote the first paragraph of the Discussion to explicitly describe these two important aspects of modeling used in our work.

Line 29: The authors state: “Analysis reveals that ferritin and iron regulatory protein regulation are the main drivers of mucosal block, secondary to dose magnitude and regulation by hepcidin.” How can they be the main drivers if they are secondary to dose magnitude and regulation?

Thanks for pointing out this apparent contradiction in the text, we have now better described this as follows:

“Analysis reveals that ferritin and iron regulatory proteins are the main intracellular mechanisms contributing to the mucosal block, findings congruent with experimental predictions”

Given that our manuscript is dedicated to studying the intracellular contributions to the mucosal block it is better to not refer to the systemic drivers in the abstract. We thank the reviewer for this, as it helps us focus the text towards what is really studied in this manuscript.

Line 373: they should use an equation editor to present those equations. Also, I don't think they define R…

We have now typeset the equations in a clearer way. We also corrected the mistake: it is indeed R that is defined in line 373

line 390: Table 2 should include units for each entry.

Following the definition given in line 373, these quantities are adimensional, they represent ratios of relative changes (relative changes already have no units, and neither does their ratio).

Line 71: "there has been a distinction between the mucosal iron absorption block mediated by hepcidin and a local intestinal block that is dependent on the biochemistry of individual enterocytes...In this work, we use computational modeling to estimate the quantitative impact of distinct cellular mechanisms within individual enterocytes ..." This sounds like the effects of hepcidin are not considered in their model, but in the rate-law for FPN inactivation, the Fe concentration in the blood is a factor, which suggests that hepcidin is indirectly being considered. This should be clarified.

Thank you for pointing this out; indeed our model is not intending to identify the role of hepcidin regulation (a systems level negative feedback roughly proportional to the level of whole body iron), but hepcidin regulation is always present so we also have to include it in the model. In order to make this explicit, we added the following sentence to the end of that paragraph in the manuscript:

"While our focus is on the cellular mechanisms, still the systemic effect of hepcidin has to be considered as enterocytes *in vivo* are always affected by it."

I believe that iron-loaded enterocytes (when FPN has been inactivated) are sluffed off the intestinal lining and exit the body, and that this prevents iron absorption. Shouldn't this relevant process be included in their model?

The process of sloughing is involved in tissue-level dynamics, while the present model is for enterocytes alone. As we mentioned, replying to a similar point made by reviewer #1, creating a dynamic model at tissue-level model would be a much larger project which would at least double the size of this manuscript. We would like to study that problem and plan to do so in a follow-up project, so it will have to come as a later publication. Note that this is a more complex type of simulation (multiscale) which will require combining ODEs with agent-based simulation, including the spatial arrangement of cells and PDEs for certain signaling molecules that diffuse.

In order to express this in the manuscript, we added a paragraph to the discussion:

"The high buffering capacity of FT also has consequences at the tissue-level given that duodenal enterocytes have short life span and are sloughed to the intestinal lumen at a considerable rate [104]; thus it is expected that such sloughing of enterocytes with high ferritin-bound iron content will result in irreversible loss of iron that had been uptake by enterocytes but will be irreversibly lost from being transferred to the rest of the body. To investigate such dynamics, however, is beyond the scope of this work and will require a multiscale model of the villus structure and dynamics."

Line 274: ..."the more sensitive the degradation of FPN is to the level of hepcidin, the smaller the amount of iron transferred to the plasma is". I would think that the more sensitive the process is to hepcidin, the more sensitive the cell controls the export of iron to the plasma.

No, enterocyte cells have no direct control over the amount of hepcidin in the blood; they passively respond to hepcidin, with a decrease of the rate of export of iron. Hepcidin is controlled by other organs (mostly the liver). The sensitivity of FPN degradation to hepcidin is complex process that involves ubiquitination and proteasome degradation of FPN-hepcidin complex. So far that process does not seem to be regulated by any of the variables in the enterocyte model (LIP and IRPs are not involved in this process).

Do mammalian cells in culture (e.g. Caco-2 cells) exhibit the mucosal block phenomenon? Or is this something only observed in the intestines of mammals? The answer will impact the utility of using literature results involving treatments of mammalian cells in culture with iron.

The short answer is that yes they do, as shown by our ref. [19] (Sharp et al. 2002 510:71–76. doi:10.1016/s0014-5793(01)03225-2). However, even if these cells did not display mucosal block they would still be useful in providing data for estimation of several model parameters. Importantly, that while we used *in vitro* Caco-2 data to calibrate the model, we validated the resulting model parameters with *in vivo* data, where both data and model displayed the iron block.

Reviewer #3

In https://doi.org/10.1186/1752-0509-4-147 it was presented a model that "predicts desirable characteristics for a buffer protein such as effective removal of excess iron, which keeps intracellular cLIP levels approximately constant even when large perturbations are introduced, and a freely available source of iron under iron starvation. In addition, the simulated dynamics of the iron removal process are extremely fast, with ferritin acting as a first defense against dangerous iron fluctuations and providing the time required by the cell to activate slower transcriptional regulation mechanisms and adapt to iron stress conditions." Please correct some claims in the manuscript accordingly.

We agree with the reviewer that ferritin does have a role in limiting the spiking of LIP as pointed out in the quoted text from Salgado *et al.* 2010. What our results indicate is that the internalization of DMT1 also strongly contributes to the protection of LIP excess, as seen from the results in our Fig. 5. We've updated the text to mention the recognized importance of ferritin in this context as well, and cite Salgado et al. 2010. Our text in the (lines 552-553) now reads:

"Faster rates of endocytosis serve to limit the transient peak of intracellular LIP (cellular free iron concentration) after an iron dose (Fig. 5F). This effect is additional to the previously identified role of FT in buffering LIP levels [105]."

There is no discussion regarding the relative characteristic time of each phenomenon considered in the model and its impact on the construction of the model and subsequent conclusions. This is relevant considering the long simulation ranges up to 72 h and the different regulation systems that are in place: systemic, transcriptional, translational and mucosal block, with response times varying from days to minutes after an iron challenge. Hepcidin regulation is considered, but DMT1 and FPN regulation are not included in the model. This could have a strong impact on the results of the model, given the long simulation times.

Indeed longer time scales could bring in other levels of regulation. However in this case we know from Gunshin *et al.* 1997 [38] that DMT1 is not significantly transcriptionally regulated in mature enterocytes (at the tips of the villi), and from Galy *et al.* 2013 [34] that DMT1 is also not positively regulated by IRPs in the intestine of adult mice. Furthermore, Galy et al. 2013 [34] also show evidence that FPN activity is not so dependent on IRPs and is likely mostly regulated by hepcidin. Since our validation covered this long experiment/simulation times we expect these effects to be small (otherwise they would have caused our simulation to not match the validation

experiments)

The data from Esparza (2015) indicate that the kinetics of DMT1 endocytosis are rapid and vary depending on the type of iron administered. The simulations presented in Figures 2B and 2D do not adequately represent the complexity of the data from the original studies. Specifically, in Figure 2B, the model depicts an exponential decrease in the amount of DMT1 on the membrane that continues for up to 120 minutes of simulation. However, the data from Esparza (2015) show a reappearance of DMT1 on the apical membrane after 20 minutes, with the rate of reappearance depending on the type of iron.

Figure 2B includes data from Esparza 2015 [27] *and* from Ma 2002 [28] (where we used data from their Figure 3, sections 1, basal, and 12, apical). Note that both show the same trend, even though came from completely independent labs. When both data sets are overlayed (our Fig 2B), it is clear that the underlying behavior is a monotonic decrease. While the data from Ma do not include error bars, the data from Esparza do and they are fairly large, thus both behaviors could be possible, however the simpler one is what our fit converged to. Additionally, while the experimental data of Esparza could be interpreted to display oscillations, the model adopted in Esparza does not, and in fact also displays a monotonic decrease like our model (see Esparza's Fig. 8)

The data from Colins (2017) indicate that the displacement of transporters or mucosal block can occur in less than 30 minutes. The experimental results reveal changes in the slopes of absorbed iron that the model does not account for. In fact, the model presents a linear approximation of iron trajectories, which may be an oversimplification. This is evident even in sequences as short as 8 to 10 minutes: "Between 3 and 12 min after the iron exposure, there is a significant decrease in the rate of iron absorption compared to its initial value. Nevertheless, during the next five minutes, the rate increases again. This behavior is observed for the three initial apical iron concentrations. The experimental patterns observed in the absorption rates over time for the three extracellular iron con-centrations studied drift away from the standard behavior of a transport system that could be described using a Michaelis–Menten or Hill type of expression. This behavior can be attributed o the variation in the amount of DMT1 present in the apical membrane after the iron expo-sure, as suggested by Nuñez et al"

We emphasize that this issue is about validation of our model, not its calibration. Our Figure 2D includes data from Collins 2017 [30] and Cegarra 2019 [14]. The issue here is similar to the previous point regarding the kinetics of DMT1 endocytosis. While the data in Collins' Fig 2 may be interpreted as a complex trajectory, in fact the error bars are large enough to raise doubt about the non-monotonicity. Smooth monotonic curves also fit the data equally well and carry less assumptions.

In order to fit the varying rates in the trajectory of apical iron uptake, the model in Cegarra adopted a cycling of DMT1 such that in 15 minutes it would have to cycle twice between apical membrane and endosomes, for which we could not find experimental data backing this. However, we emphasize that this Figure is showing *validation* of our model, and as a fact, its behavior approximates the data well enough for us to be satisfied. (We would prefer not to increase the complexity of our model without sufficient data to calibrate it properly, and if we used these data to calibrate, then we would have no way to validate the model, after all.)

Based on this statements such as "Without modifying any parameters, other than the initial conditions reflective of each experiment, the model adequately reproduced the observations of the validation experiments (Fig. 2D-F)." may not be fully accurate..

We are not sure what the reviewer is expressing here. The reproduction of the data in figures 2D-F definitely only required changing the initial conditions. Is the reviewer saying that the model did not adequately reproduce the observations? As argued in the previous comment, we think the data displayed in the figures shows that it

does, particularly considering that the model was calibrated exclusively with data in panels A-C (180 minutes, *in vitro*) and the data E-F covers 0-72 hours (including *in vivo*, panels E and F). The manuscript also discusses these issues in the paragraph starting line 204 ("The model generally matches the experimental observations …"). We want the reader to be aware of these issues.

Line 212 Why is the steady state calculated in this manner? Is a stationary state of this kind reasonable from a biological perspective, given that the availability of iron in the apical compartment is rarely constant? Additionally, other regulatory systems operate on different time scales, and these are not accounted for in the model.

Unfortunately there is no data (to our knowledge) of *in vivo* iron concentrations in the duodenal lumen of well-fed animals. Thus it is hard to know to what extent are the temporal oscillations in its concentration due to meals (including issues of meal frequency, gastric emptying speeds, microbiome, etc.).

A steady state requires that the external species be constant and it is also asymptotic; we simulated for a large time in order to allow the system to settle. Of course the reviewer is right that there is never a steady state in the intestine given regular meals. However for all the time course simulations that we carry out it is important to have initial conditions that are in the operating *range* for that physiology (otherwise the transients would be "contaminated" with the uncertainty of the initial conditions). Note that the rest of the manuscript describes simulations that are not in a steady state, these three steady states are only used as initial conditions for the time course simulations. We added the following text to the section on steady states to make this more explicit:

"While enterocytes are never in a steady state, given the perturbations caused by meals, it is useful to establish a steady state that would exist if the average iron from meals was continuously infused. Such steady states provide the average values of each variable and will be used as initial conditions for time courses."

Line 219 Please explain the rationale for choosing a concentration of 125 nM in the intestinal lumen and why it should be so high to indicate iron overload.

The doses chosen were based on experimental ranges reduced to the scale of one cell. The "high" iron dose is a 10-fold higher concentration than the "adequate" iron dose.

Line 252 Including the experimental data in the figure would help evaluate the simulation results more effectively. Fig. 6 of Frazer et al. 2003 [15]

To answer this issue we now include a section in the Supplement S1, further comparing our model's output to the experimental results in Frazer *et al.* 2003 [15], beyond what is shown in the main manuscript. These results back up our claim that "The magnitude of the simulated difference in block between the lowest and highest dose – around 3.5-fold for uptake (Fig. 3A), and 2-fold for transfer (Fig. 3C) – as well as the linear relationship between dose size and block depth (Fig. 3E) agree with experimental data (also linear with 4-fold block increase from loading dose variation, see Fig. 6 of Frazer et al. 2003 [15])."

Line 329 The manuscript presents numerical simulations illustrating the effects of Kcat in LIP-induced DMT1 endocytosis and Kcat and Km in the synthesis of FT. However, it does not provide a mechanistic explanation based on the structure of the mathematical model or the behavior of the biological system. Please improve the critical discussion..

We do not understand this request very well, because after the first sentence of this paragraph, the rest (line 306 of revised ms) is an explanation of the biological meaning of the simulation:

"This rate constant represents the sensitivity of endocytosis to LIP, in other words how much DMT1 is sequestered into the cytoplasm from the apical membrane relative to the same LIP change. A higher value of this

rate constant causes more endocytosis for the same amount of LIP change. A higher value of this rate constant leads to a higher maximum reduction in iron uptake (Fig. 4A), while the other block characteristics stay essentially unchanged (Fig. 4E,I,M). This means that variations in the rate of DMT endocytosis significantly alter iron uptake by the enterocyte, but do not affect how much iron is subsequently transferred to the plasma, after being taken up by the enterocyte from the lumen. In other words, the block severity seen in iron transfer to plasma is not explained by DMT1 endocytosis."

The same happens in the section on FT Synthesis (starting line 336).

Line 297 There is a maximum in the Minimum Transfer Value plot Fig4N. Please discuss and explain why.

We thank the reviewer for this question because the answer is not trivial. However, before we explain the causes of that maximum, we highlight that *we don't think this has strong physiological significance* (after all it is a very small effect) and thus we prefer not to discuss it in the manuscript.

There are two processes co-occurring with FT iron sequestration that are responsible for the existence of a maximum in the curve of "minimum iron transfer" as a function of FT expression level.

The details of the experiment/simulation are important: First an initial iron dose is applied, followed by a variable waiting time and then a second test iron dose. The transfer is measured after the second dose. However, the first dose is important too, as well as the timing of the second dose (with the minimum transfer of the left side of the maximum in the curve of "minimum iron transfer" occurring around 55,000 s or roughly 15 hours). Since we are changing the expression rate of FT and second dose timing, the maximum observed in Fig. 4N happens at a value of FT expression that results in FT being less able to buffer the LIP accumulated in the first dose, so there is a little more LIP when the second dose happens. This, in turn, results in a lower amount of active DMT1 at the time the second dose is given (since LIP drives DMT1 down). Another mechanism that is happening simultaneously is that at lower FT expression there is higher iron transfer during the first phase (between doses) which increases FPN inactivation (through hepcidin). Both these effects operate in the first half of the curve in Fig 4N at lower FT expression values causing a slight increase in the "minimal iron transfer" as expression rate increases, however at some point the amount of FT becomes large enough that its buffering effect starts dominating and then the curve starts decreasing considerably.

In other words, there is a value of FT expression rate where the competing mechanisms are balanced yielding a maximum in the "minimum transfer value". Ferritin is 1) just high enough to limit transfer *before* the second dose happens, so FPN deactivation is minimal, and 2) also just high enough that buffering capability of LIP is such that DMT1 is not yet sufficiently affected to cause a sufficient inhibition on transfer, while 3) it is also just low enough that its buffering capacity is not yet dominating.

The 3 plots below show what happens in the time courses at expression rates close to the maximum (0.4 pMs^{-1} below the maximum, 0.63 pMs^{-1} the maximum, and 0.8 pMs^{-1} beyond the maximum)

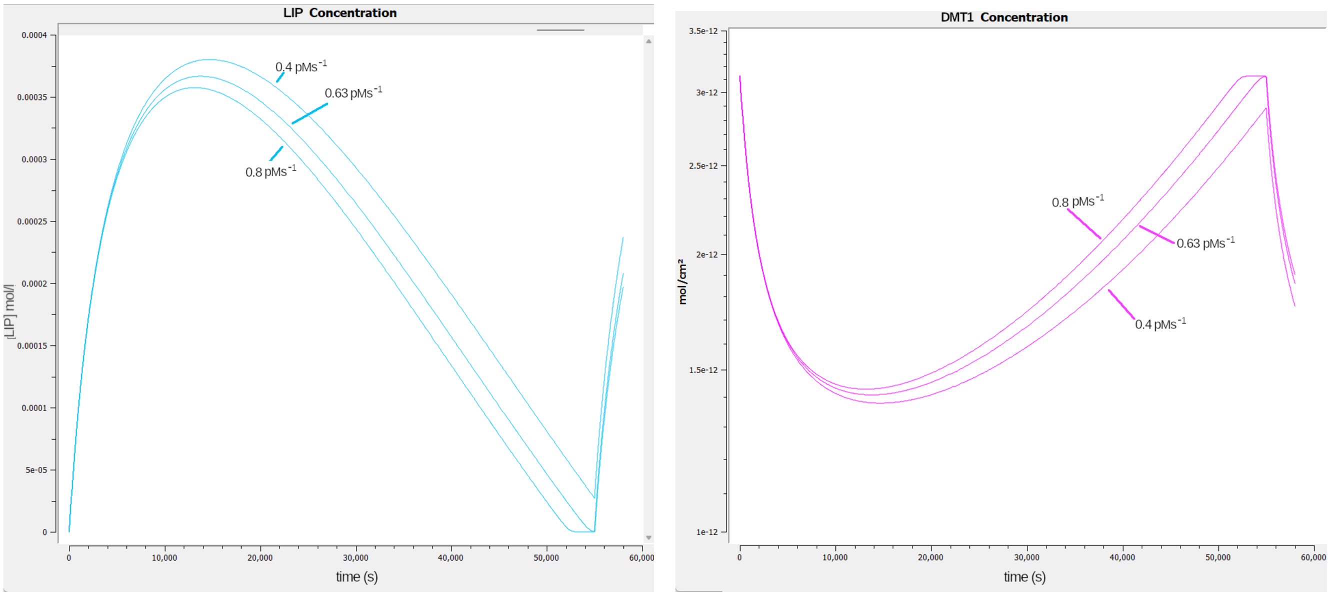

Time course of LIP and DMT1 (active) concentrations at three rates of FT expression around the maximum.

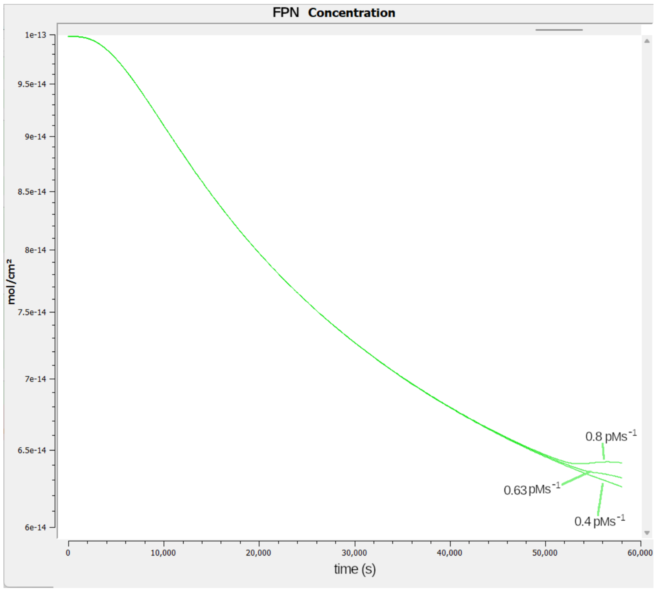

Time course of FPN (active) concentration at different FT expression rates around the maximum.

All of these of these behaviors are discussed and analyzed in the manuscript independently. However, as we mentioned above, we feel this is unlikely to be physiologically relevant given the small effect and since it may depend on the exact parameter values used in the model. As such we prefer not to discuss this maximum in the manuscript.

Line 412 Why a 12 h is chosen as the fixed-dose interval ? please explain in the manuscript.

We used this time interval as the experiments in Frazer et al. 2003 (our Ref. 15; doi:10.1136/gut.52.3.340) show the maximum block happens around 12 hours.

Lines 457-495 The discussion section requires substantial enhancement. Specifically, the content situated between these lines resembles an extended summary rather than a critical analysis of the

paper's findings.

It is true that these two paragraphs summarize the results, but the paragraphs below starting line 496 provide our critical analysis (now line 523 in the revised manuscript).

Line 479 Figure 2 illustrates that the model was calibrated using data within the range of 0 to 180 minutes. However, most of the simulations and conclusions are based on a range of 0 to 72 hours. Please discuss the impact of this discrepancy on the quality of the model and the relevance of the simulation results and conclusions.

The reviewer is correct to point out that the model was calibrated with in vitro data in a time scale of up to 3 hours. The model works on up to 72 hours. Indeed this would raise questions if we had not carried out validations in this time range (Fig 2E-F). Indeed these validation experiments are not only important by showing the model behaves correctly in this time range, but also that these experiments were carried out in vivo. This provides a good deal of confidence that the model can be used for such time scales.

Line 652: Additional reactions are mentioned; however, the equations and parameters are not provided. Please include all differential equations, initial conditions, and model parameters in a mathematical format that is not dependent on the COPASI platform in the supplementary material.

We have added an extra table showing the additional reactions. We also added all the differential equations of the model in a new supplement PDF.

Line 374 possible typo: R instead of epsilon

Thank you for pointing this out, we have corrected to R

Line 417 possible typo: 48 instead of 24? At 24 hours, the body's iron is increasing, and the LIP has a substantial amount of iron.

Thank you for spotting this, it is indeed a typo and should be 48 hours; the text has been corrected.

Line 626 possible typo: space between FP_N

Thank you for pointing this out, we have corrected the typo
